# DMLS: an automated pipeline to extract the *Drosophila* modular transcription regulators and targets from massive literature articles

**DOI:** 10.1093/database/baae049

**Published:** 2024-06-20

**Authors:** Tzu-Hsien Yang, Yu-Huai Yu, Sheng-Hang Wu, Fang-Yuan Chang, Hsiu-Chun Tsai, Ya-Chiao Yang

**Affiliations:** Department of Biomedical Engineering, National Cheng Kung University, No.1, University Road, Tainan 701, Taiwan; Medical Device Innovation Center, National Cheng Kung University, No.1, University Road, Tainan 701, Taiwan; Department of Biomedical Engineering, National Cheng Kung University, No.1, University Road, Tainan 701, Taiwan; Department of Information Management, National Central University, No. 300, Zhongda RD., Zhongli District, Taoyuan 320, Taiwan; Department of Information Management, National Central University, No. 300, Zhongda RD., Zhongli District, Taoyuan 320, Taiwan; Department of Information Management, National Central University, No. 300, Zhongda RD., Zhongli District, Taoyuan 320, Taiwan; Institute of Information Management, National Yang Ming Chiao Tung University, No. 1001, Daxue Rd., East Dist., Hsinchu 300093,Taiwan

## Abstract

Transcription regulation in multicellular species is mediated by modular transcription factor (TF) binding site combinations termed *cis*-regulatory modules (CRMs). Such CRM-mediated transcription regulation determines the gene expression patterns during development. Biologists frequently investigate CRM transcription regulation on gene expressions. However, the knowledge of the target genes and regulatory TFs participating in the CRMs under study is mostly fragmentary throughout the literature. Researchers need to afford tremendous human resources to fully surf through the articles deposited in biomedical literature databases in order to obtain the information. Although several novel text-mining systems are now available for literature triaging, these tools do not specifically focus on CRM-related literature prescreening, failing to correctly extract the information of the CRM target genes and regulatory TFs from the literature. For this reason, we constructed a supportive auto-literature prescreener called *Drosophila* Modular transcription-regulation Literature Screener (DMLS) that achieves the following: (i) prescreens articles describing experiments on modular transcription regulation, (ii) identifies the described target genes and TFs of the CRMs under study for each modular transcription-regulation-describing article and (iii) features an automated and extendable pipeline to perform the task. We demonstrated that the final performance of DMLS in extracting the described target gene and regulatory TF lists of CRMs under study for given articles achieved test macro area under the ROC curve (auROC) = 89.7% and area under the precision-recall curve (auPRC) = 77.6%, outperforming the intuitive gene name-occurrence-counting method by at least 19.9% in auROC and 30.5% in auPRC. The web service and the command line versions of DMLS are available at https://cobis.bme.ncku.edu.tw/DMLS/  **and**  https://github.com/cobisLab/DMLS/, respectively.

**Database Tool URL**: https://cobis.bme.ncku.edu.tw/DMLS/

## Introduction

Gene transcription regulation in multicellular species is mediated by *cis*-regulatory elements in a modular manner (1). These modularized *cis*-regulatory elements are collectively termed *cis*-regulatory modules (CRMs). CRMs are usually composed of several transcription factor (TF) binding sites (TFBSs) and can cooperate with other chromatin-binding proteins to alter the expression patterns of their target genes (2). Diverse CRM functions and the participating regulators have been explored in the literature. For example, researchers found that the failure of specific regulator binding events on CRMs caused by single-nucleotide mutations in the CRM sequences can lead to carcinogenesis (3). Therefore, CRMs and their targets have been investigated extensively in recent transcriptional regulation studies.

The participating TFs, their binding sites and the target genes in modular transcription regulation are usually identified via reporter assays and mutational analysis (2). Nevertheless, the CRM experimental results of their participating regulators and targets are mostly fragmentary throughout the literature. Researchers must spend a lot of time surfing through the articles deposited in biomedical literature databases such as PubMed to obtain the information (4). However, with the advancement of science, more and more articles are deposited in the literature databases. For example, it is estimated that PubMed indexed more than 5692 *Drosophila*-related papers in 2023. This rampant growth of scientific articles makes the situation even worse for knowledge sharing of studies in modular transcription regulation (5).

Currently, only the REDfly database (6) deals with the data fragmentary issue on CRM regulators and target genes in *Drosophila*. REDfly stores the manually curated CRM-related articles and the CRM target genes described in these articles. It also gathers experimentally verified TFBSs. Thanks to these great efforts, many CRMs, their target genes and the involved potential regulators can be queried and explored in one database. Nevertheless, these laborious curation processes require substantial human resources to keep up with the rapid growth of scientific research papers (7). Novel text-mining systems have been developed with the aim of speeding up the literature triaging step and lowering the burdens on human curators (8). Among them, several rule-based and co-occurrence-based text-mining tools were implemented to help the relation-mining task (9). However, these tools focus on extracting the grammatical noun pairs within the same sentences. They cannot specifically apply to the task of literature screening for the described regulators and target genes of a CRM studied in an article. Previously, we have developed a tool called YTLR (4) that helps comprehend yeast transcription-regulation articles. Nevertheless, genes are regulated in a more switch-like manner in yeast, and YTLR was developed to extract the switch-like TF-gene binding/regulatory pairs from only two consecutive sentences. Since most CRM-related articles for metazoan species describe only the CRM target genes in a paragraph, the switch-like TF-gene extraction pipeline design in YTLR fails to be well-suitable for comprehending modular transcription-regulation-related articles and cannot recognize CRM target genes studied in these CRM-related articles. Because of these issues, we need an automated pipeline that meets the following requirements to facilitate the CRM experiment result collection: (i) provides rapid prescreening to identify articles related to modular transcription regulation, (ii) identifies the described target genes and regulators of CRMs under study from the identified modular transcription-regulation-related articles and (iii) features an automated interface to perform the task. These prescreening functions can help accelerate the curation process and support the construction of customized knowledge bases. Nonetheless, no existing tool has satisfied the aforementioned goals until now. Hence, there is still a need to develop an automated pipeline to support and speed up the gathering of knowledge of previous CRM experimental results.

In this research, we built an automated pipeline called *Drosophila* Modular transcription regulation Literature Screener (DMLS). DMLS aims to facilitate the literature triaging step for the described target genes and regulatory TFs of the CRMs under study from each CRM-related article. It mainly focuses on prescreening and generating customized in-house CRM knowledge bases in *Drosophila*. DMLS is an automated literature reader pipeline consisting of deep learning models. In DMLS, articles that study modular transcription regulation are first recognized from the abstracts downloaded from PubMed. Then, for these CRM-related articles, the described target genes and regulatory TFs for the CRMs under study are identified. We verified that the performance of the DMLS tool in identifying the literature related to modular transcription regulation achieves test area under the ROC curve (auROC) and area under the precision-recall (PR) curve (auPRC) values of 92.4% and 94.1%, respectively. Moreover, DMLS can identify the described TF and target gene lists of CRMs under study in CRM-related articles with the macro test auROC and auPRC of 89.7% and 77.6%, respectively. These results outperform the intuitive occurrence-counting baseline method by 19.9%/30.5% in auROC/auPRC. Finally, a walk-through case study is provided to demonstrate the utility of DMLS in accelerating the literature triaging steps. In this example, we further show that DMLS can be easily coupled with CRM analysis tools to help delve further into the CRM sequences and functions. To facilitate the usage of DMLS for users in building their private CRM knowledge bases, a command-line interface and a web service were implemented. The DMLS web service and the command line version can be accessed and downloaded at https://cobis.bme.ncku.edu.tw/DMLS/ and https://github.com/cobisLab/DMLS/, respectively.

## Materials and Methods

### The workflow of the DMLS pipeline

**Figure 1.**
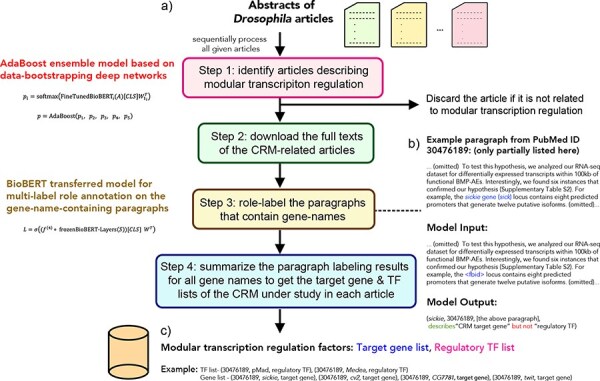
The workflow of the DMLS pipeline. (a) Step overview of the pipeline. (b) The example case for DMLS Step 3. (c) The example case for DMLS Step 4.

The DMLS pipeline is designed to serve as a literature prescreener and knowledge extractor for CRM regulators and target genes. The overall workflow of DMLS is depicted in Figure 1. Users need to input the abstract .csv file downloaded from PubMed. This .csv file can contain one or more abstracts of interest. Users can specify DMLS to choose only the articles that include the keyword *Drosophila* or fly in the title or abstract. Then, DMLS will process these abstracts one by one. For every article, DMLS performs the following four steps to extract the described CRM regulator and target gene information (Figure 1): (i) Step 1: identify articles describing modular transcription regulation. The given abstract is fed into a deep learning model to check if it is a CRM-related article. (ii) Step 2: download the full texts of the CRM-related articles. For an identified CRM-related article, DMLS will download its full text using the PubMed Central File Transfer Protocol (PMC FTP) service if possible. When no freely available full text can be found for an article, DMLS will consider only its abstract in Step 3. (iii) Step 3: check and label the gene name–containing paragraphs in the identified CRM-related articles. In this step, DMLS comprehends the paragraphs that contain at least one gene/protein name in a per-name manner to see if these gene names are the described CRM target genes and/or regulatory TFs in the paragraphs. (iv) Step 4: summarize the target gene and regulatory TF lists for the CRM-related articles. The final outputs associate each PubMed ID with the described target gene and TF lists of the CRMs under study as a customized knowledge base.

The outputs of each step in DMLS are explicitly stated as follows: DMLS Step 1 reads the abstract of different papers and sifts out the articles describing *Drosophila* modular transcription regulation, resulting in PubMed IDs for CRM-related articles. After that, DMLS Step 2 helps download the full texts of the identified CRM-related articles using the PMC FTP service, resulting in the .xml files that contain the full texts of these CRM-related articles. In DMLS Step 3, paragraphs describing TFs and target genes participating in the CRMs under study are comprehended by a fine-tuned deep network. The gene names, TF/target gene labeling of these gene names in the paragraphs and the paragraphs supporting the labeling decisions are provided after DMLS Step 3 for users to further read the detailed regulation mechanism. Finally, DMLS Step 4 summarizes the paragraph comprehension results into lists. Therefore, for a given article with its PubMed ID, users will end up with a target gene list regulated by a TF list in a modular manner as the final output. The design details and the datasets prepared for fine-tuning the deep networks are articulated in the following subsections.

### DMLS Step 1: identifying articles related to modular transcription regulation

In this step, articles that describe experiments on modular transcription regulation (i.e. CRMs) are identified via a deep ensemble model. To achieve this purpose, we first collected an article ground-truth dataset consisting of CRM-related and non-CRM-related articles. Then, based on the article ground-truth dataset, we trained a deep ensemble model that helps distinguish the articles related to modular transcription regulation from non-CRM-describing articles based on their abstracts.

#### Collecting the article ground-truth dataset

We prepared an article ground-truth dataset to train and evaluate the DMLS Step 1 deep ensemble model. Generally, the training and evaluation processes require positive samples (articles describing modular transcription regulation in this case) and negative samples (articles that do not provide transcription regulation information in this case) to form the ground-truth dataset (10, 11). We downloaded 1015 manually curated CRM-related abstracts from REDfly (6) as positive samples. In order to prepare the negative samples, we gathered 101 760 *Drosophila* articles published between 1985 and 2020 that were not curated by REDfly to have CRM information. Since the positive and negative sample numbers are quite imbalanced, we designed a bootstrap ensemble model to handle this data number imbalance issue (12, 13). In order to support the training and evaluation of the designed ensemble model, the article ground-truth dataset was divided into three parts: the training-validation set, the ensemble set and the test set. Publications before 2014 were put into the training-validation set, and articles published during 2014–6 were included in the ensemble set. For articles published after 2016, they were reserved in the test set. In total, 630 articles describing modular transcription regulation were used in the training-validation set, 286 CRM-related articles were in the ensemble set, and the remaining 99 CRM-related articles were reserved in the test set. To prepare the negative samples for the ensemble set and the test set, we sampled 285 articles published during 2014–6 and 99 articles indexed after 2016, respectively, from those articles that REDfly did not mark as having modular transcription regulation information.

#### CRM literature identification deep networks

Since the article ground-truth dataset suffers from immense positive–negative data number imbalance, we designed a data-bootstrapping ensemble model to handle this issue in DMLS Step 1 (Figure 1a, Step 1). The ensemble model consists of two parts. In the first part, five data-bootstrapping base networks were trained to leverage the variations among the large negative samples. In the second part, these five data-bootstrapping base networks were summarized using an AdaBoost model.

It is known that the ensemble approach can compensate for the biases of individual models to improve the overall identification performance (4, 13–15). To compensate for the variation of the massive negative samples in our article ground-truth set, we obtained five data-bootstrapping networks and applied the AdaBoost algorithm to aggregate the final ensemble results. The variation of the negative samples is captured by these five data-bootstrapping networks trained on different sampled negative subsets. To achieve this goal, we first sampled five different negative subsets, each with 630 negative samples, from the negative set articles published before 2014. These five sampled negative subsets were separately paired with the 630 CRM-related articles published before 2014 to form five bootstrapping datasets. Based on these five bootstrapping datasets, we fine-tuned the Bidirectional Encoder Representations from Transformers for Biomedical Text Mining (BioBERT) pretrained model (16) to obtain five data-bootstrapping networks using 5-fold cross-validation. We added an extra linear layer after the BioBERT [paragraph encoding vector (CLS)] encoding to transfer the pretrained BioBERT model into our DMLS Step 1 usage:


$${P_i} = {\mathrm{softmax}}\left( {{\mathrm{finetuned\_BioBER}}{{\mathrm{T}}_{\mathrm{i}}}\left( A \right)\left[ {{\mathrm{CLS}}} \right]W_{{1_i}}^T} \right),$$

where *A* is the word piece tensor for the given abstract, $W_{{1_i}}^T$ is the trainable weight matrix for the *i*th bootstrapping dataset, $\mathrm f\mathrm i\mathrm n\mathrm{et}\mathrm u\mathrm n\mathrm e\mathrm d\_\mathrm B\mathrm i\mathrm o\mathrm B\mathrm E\mathrm R{\mathrm T}_{\mathrm i}\left(A\right)\left[\mathrm C\mathrm L\mathrm S\right]$ refers to adopting the CLS encoding vector after the BioBERT computation and ${P_i}$ is the probability estimated by the *i*th data-bootstrapping network that the given article describes modular transcription regulation information. This same deep learning network architecture was separately applied to the five data-bootstrapping datasets. Data variation lying within the ground-truth dataset was leveraged through these data-bootstrapping networks. After training the five data-bootstrapping networks, the Adaboost algorithm was used to aggregate the final prediction:


$$P = {\mathrm{AdaBoost}}\left( {{P_1},{\mathrm{\;}}{P_2},{\mathrm{\;}}{P_3},{\mathrm{\;}}{P_4},{\mathrm{\;}}{P_5}} \right),$$

where ${P_i}$ is the estimated probability by the *i*th data-bootstrapping network and *P* is the final aggregated probability that the article describes modular transcription regulation. The AdaBoost algorithm was optimized by the 5-fold cross-validation and learning curve methods on the ensemble set. In summary, to identify if a given *Drosophila* article describes CRM-related information, the abstract is screened by five data-bootstrapping networks followed by the AdaBoost aggregation to obtain the final prediction probability.

#### DMLS Step 1 model hyperparameters

We selected the hyperparameters of the five data-bootstrapping base networks and the final ensemble AdaBoost model by 5-fold cross-validation. The following hyperparameters were selected for the five data-bootstrapping base networks: (i) learning rate schedule: cosine decay (max learning rate = 5 × 10^−6^); (ii) optimization method: Adam; (iii) neuron initialization: pretrained BioBERT; (iv) number of epochs: 3 since we were fine-tuning the BioBERT weights; (v) loss function: categorical cross-entropy; (vi) batch size: 16; and (vii) dropout rates: 0.1 and 0.4 for the dropouts in the encoder part and the last fully connected layer, respectively. These dropout values were added to prevent overfitting. For the AdaBoost ensemble model, the following hyperparameters were used: (i) base learner: 150 decision stumps with max depth 2 and (ii) learning rate: 0.2.

### DMLS Step 2: retrieving the full texts of the identified articles that are related to modular transcription regulation

DMLS retains only those articles that describe modular transcription regulation experiments after Step 1. For these identified CRM-related articles, DMLS then downloads their full texts from the PMC database. The PMC database collects the charge-free full texts of biomedical papers. Since web crawling on the PMC website is not permitted, we implemented a download procedure that utilizes the PMC FTP service to retrieve the available full texts of these CRM-related articles. If no full text for some CRM-related article is available, the abstract of it will be used in Step 3. After this step, the full texts or abstracts of the articles related to modular transcription regulation are submitted to DMLS Step 3 and then Step 4 for extracting the described target genes and regulatory TFs in the studied modular transcription regulation.

### DMLS Step 3: labeling the roles of the described gene names contained in paragraphs of CRM-related articles

In this step, DMLS tries to comprehend the described roles of the gene names contained in the paragraphs of articles identified to report modular transcription regulation experiments. DMLS Step 3 first parses out the paragraphs that contain at least one gene/protein name. Then, for every gene name, along with the paragraph where it appears, DMLS utilized a deep network to comprehend the regulatory role conclusions (target gene or regulatory TF) that the paragraph provides for the gene name. The deep comprehension network in DMLS step 3 is trained based on a prepared gene name–paragraph pair ground-truth dataset.

#### Preparing the ground-truth dataset of gene name–paragraph pairs

In REDfly, experts curated CRM information in *Drosophila* from 1015 CRM-related articles. Among them, the full texts of 419 papers are freely available in the PMC database and contain the description wordings of the reported target genes. In this research, we only used the paragraphs from these 419 full texts for DMLS Step 3 and Step 4. The dataset used in DMLS Step 3 is the collection of paragraphs that include at least one gene/protein name. DMLS Step 3 aims to comprehend the described roles of the mentioned gene names in these paragraphs. Thus, data in the ground-truth set of DMLS Step 3 are in the form of (gene/protein name, PubMed ID, paragraph, target gene/TF labels). These data were retrieved from the full texts of the original papers based on the information provided by REDfly. First, the full texts of CRM-related articles were parsed, and the paragraphs with at least one gene/protein name were retained. The ‘Methods and Methods’, ‘Supplementary Material’, ‘Abbreviations’, ‘Acknowledgments’ and ‘Reference’ sections were eliminated since these sections usually do not provide experimental result explanations. The synonym information of each gene/protein was downloaded from Flybase (17). Since several fly gene/protein synonyms are identical to some common English words, we manually re-scanned the list and filtered out those identical to common English words to reduce ambiguity. Second, we labeled the described roles (target genes or regulatory TFs) of every gene name in these paragraphs. If a gene name described in a paragraph was curated by REDfly experts to be the target gene of the CRMs under study for a specific article, the label of this (gene/protein name, PubMed ID, paragraph, label) pair was tagged to include ‘target gene’. To get the information on the regulatory TFs involved in the literature CRMs, we downloaded the experimentally verified TFBSs of 208 TFs from REDfly. Then, we overlapped the chromosomal regions of these TFBSs with the locations of CRMs curated from the literature. Whenever some TFBS overlaps with some CRM studied in an article, this TF is annotated as the regulatory TF involved in this CRM. If a gene name in a paragraph is the same as the involved regulatory TFs of the CRMs under study within a CRM-related article, the label of the (gene/protein name, PubMed ID, paragraph, label) pair will encompass ‘regulatory TF’. A gene/protein name in a paragraph can have both ‘target gene’ and ‘regulatory TF’ tags if both roles of it were found in the CRM literature. The (gene/protein name, PubMed ID, paragraph, target gene/TF labels) pairs are processed per paragraph for each non-duplicate gene name encountered in the CRM-related articles. Therefore, for paragraphs with multiple gene/TF names, every gene name is considered separately, resulting in different (gene/protein name, PubMed ID, paragraph, target gene/TF labels) tuples. For paragraphs with more than 512 word pieces, the upstream and downstream 256 word pieces of the observed gene name in the pair were taken. An example of the labeled gene name–paragraph pair can be checked in Figure 1b.

In total, 6885 (gene/protein name, PubMed ID, paragraph) pairs were labeled to describe CRM target genes and 3099 (gene/protein name, PubMed ID, paragraph) pairs were labeled to depict CRM regulatory TFs. Among them, 391 pairs were checked to include both labels. For paragraphs containing gene names that have no ‘target gene’ nor ‘TF’ labels, we tried to sample such negatively labeled pairs to be of the same counting number as the pairs having the ‘target gene’ label in the same article. Finally, to train the deep paragraph comprehension network in this step, we arranged the pairs from articles published before 2017 as the gene name–paragraph training–validation set. In total, 5659/2411/5586 pairs with ‘CRM target gene’/‘regulatory TF’/‘none’ labels were put in the training–validation set. The final generalization test is performed in DMLS Step 4 since the manually confirmed ground-truth CRM target gene and regulatory TF lists for each article are only provided at the article level. Therefore, we deferred the final generalization evaluation of the information extraction and summarization to DMLS Step 4.

#### Deep paragraph comprehension network for labeling the described roles of genes in paragraphs containing them

In DMLS Step 3, we constructed a deep paragraph comprehension network to help infer the described transcription roles of the gene/protein names mentioned in each paragraph. The two possible roles of a mentioned gene name in each paragraph are the target genes and regulatory TFs of CRMs, resulting in the output tuples of DMLS Step 3 in the form of (gene/protein name, PubMed ID, paragraph, target gene/TF labels) pairs. A (gene/protein name, PubMed ID, paragraph, target gene/TF labels) pair can be marked with only one label, with both labels (meaning having both roles) or with no label (meaning not related to the CRMs under study), representing a multi-labeling problem. The deep paragraph comprehension network takes a gene name–paragraph pair as its input and adopts the following architecture:


$$L = \sigma \left( {({f^{\left( 4 \right)}} \circ {\mathrm{\;frozen\;BioBERT\;layers}}\left( S \right))\left[ {{\mathrm{CLS}}} \right]{\mathrm{\;}}{W^T}} \right),{\mathrm{\;}}$$

where *S* is the paragraph word piece tensor, $f$ is the transferred transformer encoder layers in BioBERT (${f^{\left( 4 \right)}}$ means the last four layers), $ \circ $ denotes the function composition, CLS denotes the paragraph encoding vector, $\sigma $ represents the sigmoid function, $W$ is the trainable parameter matrix that maps the output tensor into a 2-D score tensor and $L$ is the final probability vector for the target gene and TF labelings. In constructing the deep paragraph comprehension network, we froze the first eight layers in BioBERT (the ‘${\mathrm{frozen\;BioBERT\;layers}}$’ function in the above formula) and fine-tuned the last four layers (${f^{\left( 4 \right)}}$ in the above formula) (18). After the deep network, the final probabilities for the target gene and regulatory TF labels of the gene name mentioned in each gene name-containing paragraph are estimated.

#### Network optimization

Since checking if the (gene/protein name, PubMed ID, paragraph) pair includes ‘CRM target gene’ or ‘CRM regulatory TF’ labeling forms a multi-labeling task, we utilized the weighted binary cross-entropy loss when optimizing the deep paragraph comprehension network. The weighted binary cross-entropy (WBCE) loss for the *i*th paragraph pair is defined as follows:


$$\begin{aligned}{\mathrm{WBCE}}\left( {{S_i}} \right) = & {\mathrm{\;}}{w_i}\mathop \sum \limits_{c = 1}^2\left( {p_c} \times {y_{c\_i}}\log \left( {{L_i}\left[ c \right]} \right)\right. \nonumber \\ & \left.+ {\mathrm{\;}}\left( {1 - {y_{c\_i}}} \right){\mathrm{log}}\left( {1 - {\mathrm{\;}}{L_i}\left[ c \right]} \right) \right),\end{aligned}$$

where ${S_i}$ is the paragraph word piece tensor for the *i*th pair, ${p_c}$ is the label-specific positive weighting for the *c*th label, ${w_i}$ is the instance-specific weighting for the *i*th paragraph pair, ${L_i}\left[ c \right]$ is the probability of the *c*th label for the *i*th gene name–paragraph pair and ${y_{c\_i}}$ is the ground-truth labeling for the *c*th label of the *i*th pair. The label-specific positive weightings (${p_c}$) were added to handle the positive/negative number imbalance issue in each labeling. The instance-specific weighting (${w_i}$) for each pair was incorporated into the training process to reduce the article length bias. In this research, the positive weightings used in training the deep paragraph comprehension network were 1.39 and 4.35 for the target gene and TF labels, respectively. The instance-specific weighting for each pair was set to be the reciprocal of the number of paragraphs describing the same gene name within the same article. Finally, 5-fold cross-validation was applied to the training process of the deep paragraph comprehension network to choose the best-fit hyperparameters.

#### DMLS Step 3 model hyperparameters

The hyperparameters of the deep paragraph comprehension network were also chosen by 5-fold cross-validation. We adopted the following hyperparameters: (i) learning rate schedule: linear warmup to the max learning rate = 5 × 5 in one epoch and cosine decay for the second and third epochs, 1e × 7 for the rest of the epochs; (ii) optimization method: Adam; (iii) neuron initialization: the best fine-tuned BioBERT from DMLS Step 1; (iv) number of epochs: 10 epochs to tune the last four layers of BioBERT weights; (v) loss function: positive-and-sample-weighted binary cross-entropy; (vi) batch size: 32; and (vii) dropout rates: 0.1 for dropouts in both the encoder parts and the last fully connected layer.

### DMLS Step 4: summarizing the paragraph comprehension results to get the lists of described target genes and regulators of the CRMs under study in each CRM-related article

Lastly, DMLS Step 4 summarizes the paragraph comprehension results and outputs the final target gene and regulatory TF lists involved in the specific modular transcription regulation described in the articles. The outputs of DMLS Step 4 are the target gene/TF lists for each article and have the form (gene/TF name, PubMed ID, target gene/TF labels). A gene name can be described in several paragraphs of a CRM-related article [i.e. in several (gene/protein name, PubMed ID, paragraph, label) paragraph pairs], and it is assigned as the target gene of the CRMs under study if at least one paragraph pair containing this gene name is labeled as ‘target gene’ in this article. Similarly, a protein name mentioned in a CRM-related article is assigned as the regulatory TF of the CRMs under study if at least one paragraph pair containing this protein name is labeled as ‘TF’ in this article. An example of the outputs of DMLS Step 4 can be checked in Figure 1c.

#### Preparing the gene-summary test set

Since no model was trained in this step, we prepared only the test set to evaluate the performance of DMLS Step 4. The CRM-related articles published after 2016 are the untouched, clean data from the previous steps. Therefore, we prepared the gene/TF summary list test set from the CRM-related articles published after 2016. In total, 69 CRM-related articles published after 2016 were checked to describe 125 (gene name, PubMed ID) pairs with the ‘target gene’ label and 59 (gene/protein name, PubMed ID) pairs with the ‘regulatory TF’ label. Among them, six (gene name, PubMed ID) pairs have both the target gene and TF labels. In order to avoid severe sample number imbalance, the number of gene names that are neither target genes nor TFs is kept similar to the number of described target genes in the same article. Hence, we sampled 125 gene/protein names that are neither categorized as target genes nor TFs within these 69 CRM-related articles. These positive and negative gene name lists, with their summarized roles in each article, form the gene-summary test set for checking the performance of the summarized final CRM target gene and regulatory TF lists by a specific tool for each CRM-related article.

### Model performance evaluation

To evaluate the performance of the deep learning models constructed for DMLS Step 1 (identifying articles describing modular transcription regulation), we resorted to the following evaluation metrics (19):


$${\mathrm{precision\; = \;}}\frac{{{\mathrm{TP}}}}{{{\mathrm{TP + FP}}}}{\mathrm{,\;Recall\; = \;}}\frac{{{\mathrm{TP}}}}{{{\mathrm{TP + FN}}}}{\mathrm{,\;}}$$

$${\mathrm{F1\; = \;}}\frac{{{\mathrm{2\;}} \times {\mathrm{\;precision\;}} \times {\mathrm{\;recall}}}}{{{\mathrm{precision\; + \;recall}}}},{\mathrm{\;TNR\; = }}\frac{{{\mathrm{TN}}}}{{{\mathrm{TN}} + {\mathrm{FP}}}}{\mathrm{ = \;1}} - {\mathrm{FPR,}}$$

where TP is the number of correctly identified modular transcription-regulation-related articles, TN is the number of correctly identified non-CRM-related articles, FP is the number of articles that are wrongly labeled as articles describing modular transcription regulation and FN is the number of missed CRM-related articles. TNR and FPR are the abbreviations for the true-negative rate and false-positive rate, respectively. These metrics were calculated by selecting the probability prediction threshold of 0.5 in DMLS Step 1. In order to estimate the intrinsic model performance, the receiver operating characteristic (ROC) curve and the PR curve for the final obtained model were also computed. The ROC curve plots the recall values versus FPRs by varying the prediction thresholds, indicating the recall value the model can achieve when the FPR is controlled. Similarly, the PR curve tracks the precision values to the recall values with various prediction thresholds, capturing the PR trade-off when controlling the number of false positives. The intrinsic model performance can be quantified using the auROC and the auPRC.

In DMLS Step 3, the outputs are the paragraph comprehension results in the form of (gene name, PubMed ID, paragraph, target gene/TF labels) tuples. Then, in DMLS Step 4, the described TFs and target genes from different paragraphs of a given article are summarized into tuples of the form (gene name, PubMed ID, target gene/TF labels), which aggregate the paragraph labeling results for an article. In other words, the results of DMLS Step 3 are presented in a per-article-paragraph manner, while the outputs of DMLS Step 4 are provided in a per-article sense. Therefore, the performance evaluation of Step 3 and Step 4 is carried out on the correctly identified (gene/protein name, PubMed ID, paragraph, label) and (gene/protein name, PubMed ID, label) tuples, respectively. Since the paragraph comprehension results of DMLS Step 3 and the summary lists from Step 4 consider the multi-labeling problem, the one-label and macro metrics should be used (19). Take the target gene–paragraph pair labeling as an example to illustrate the metrics. The one-label performance metrics were defined similar to the ones in the previous paragraph. Specifically, in the context of one-label metrics for labeling paragraphs with target gene information, TP is the number of correctly identified (gene/protein name, PubMed ID, paragraph, label) pairs with the ‘target gene’ label, TN is the number of correctly identified (gene/protein name, PubMed ID, paragraph, label) pairs that do not have the ‘target gene’ label, FP is the number of (gene/protein name, PubMed ID, paragraph, label) pairs that are wrongly labeled with the ‘target gene’ tag and FN is the number of missed pairs that should have the ‘target gene’ tag. In addition, for DMLS Step 3, to ensure better recall and specificity trade-off, the final prediction threshold was selected to be 0.7. The one-label TF labeling metrics were defined similarly. Then, the macro performance metrics summarize the one-label results:


$${\mathrm{macro\;Recall}} = \frac{1}{2}\mathop \sum \limits_{i = 1}^2{\mathrm{Recal}}{{\mathrm{l}}_i},{\mathrm{\;macro\;FPR}} = \frac{1}{2}\mathop \sum \limits_2^{i = 1} {\mathrm{FP}}{{\mathrm{R}}_i},$$

$${\mathrm{macro\;Precision}} = \frac{1}{2}\mathop \sum \limits_{i = 1}^2{\mathrm{Precisio}}{{\mathrm{n}}_i},{\mathrm{\;macro\;F1}} = \frac{1}{2}\mathop \sum \limits_2^{i = 1} {\mathrm{F}}{{\mathrm{1}}_i},$$

where F1*_i_*, Recall*_i_*, Precision*_i_* and FPR*_i_* represent the *i*th one-label F1, Recall, Precision and FPR values (*i* = 1, 2 for the target gene labeling and TF labeling, respectively). Furthermore, the macro ROC curves can be generated by considering the macro recalls and macro FPRs when the thresholds vary. The macro PR curves can be obtained similarly by considering the macro precision and macro recall values with various thresholds. In DMLS Step 4, the performance evaluation of extracting the target gene and TF lists for each article is carried out on (gene/TF, PubMed ID, label) pairs similarly using the one-label and macro metrics with a prediction threshold of 0.7.

## Results

### DMLS Step 1 identifies articles describing modular transcription regulation experiments with high accuracy

**Figure 2.**
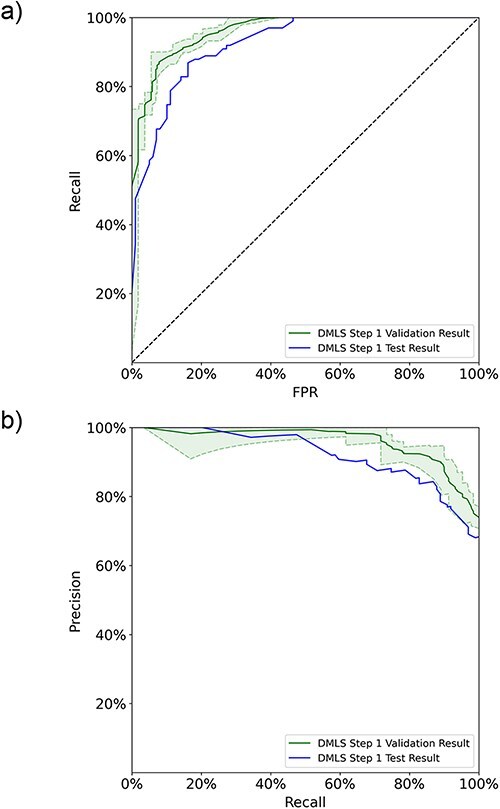
The 5-fold validation and generalization evaluation results of DMLS Step 1 on the article ensemble set and test set. (a) ROC curves. (b) PR curves. The shaded areas represent the value ranges of the cross-validation results.

In DMLS Step 1, the pipeline first distinguishes the articles describing modular transcription regulation experiments from other types of articles based on their abstracts. An AdaBoost ensemble model based on data-bootstrapping networks was constructed to achieve this purpose. The individual data-bootstrapping networks were trained on the article training-validation set using the 5-fold cross-validation and learning curve techniques to ensure that the networks were well-fitted. The well-fitting of the AdaBoost ensemble model was verified using the data number learning curves (11) and 5-fold cross-validation on the ensemble set (see Figure S1a in the Supplementary File). We collected the final cross-validation ROC and PR curves of the ensemble model on the ensemble set in Figure 2. DMLS Step 1 achieves an average validation auROC value of 94.4% and an average validation auPRC value of 93.8%. These high validation area-under-curve values verify the excellent performance of DMLS Step 1. To evaluate the generalized performance of DMLS Step 1 in recognizing articles describing modular transcription regulation, we calculated the ROC and PR curves of the final model on the reserved article test set. As shown in Figure 2a and b, DMLS Step 1 still obtains 92.3% in auROC and 92.2% in auPRC on the article test set. The close performance tracking between the validation and test results indicates that DMLS Step 1 is usable in the real world. The rest of the performance metrics are summarized in Table 1 and reveal the same trend as the auROC and auPRC values. In summary, DMLS Step 1 can accurately recognize the articles describing modular transcription regulation using their abstracts.

**Table 1.** The DMLS Step 1 (identifying articles describing modular transcription regulation experiments) performance summary of the 5-fold validation results on the ensemble set and the generalization estimation on the test set

DMLS Step 1	auROC (%)	auPRC (%)	F1 (%)	Precision (%)	Recall (%)	TNR (%)
Ensemble-validation results	94.4	93.8	86.4	86.8	86.0	87.2
Test results	92.3	92.2	83.7	84.5	82.8	84.9

### DMLS Step 3 well comprehends the described roles of the gene names in paragraphs of each CRM-related article

**Figure 3.**
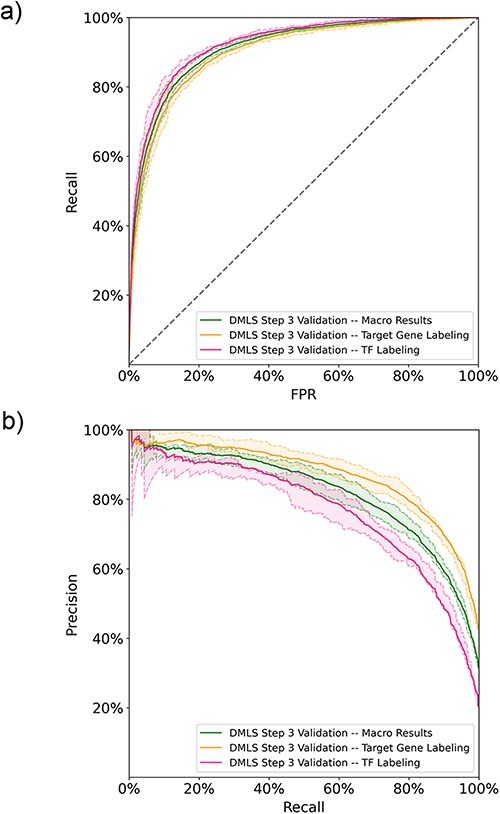
The DMLS Step 3 results of the 5-fold validation on the paragraph training-validation set computed in (a) ROC curves and (b) PR curves. The shaded areas represent the value ranges of the cross-validation results.

After DMLS Step 1 (identifying articles describing modular transcription regulation), DMLS downloaded the full texts of the identified CRM-related articles from the PMC database. Based on the downloaded full texts, DMLS Step 3 parses out the gene name-containing paragraphs and labels if they describe the target genes and regulatory TFs of the CRMs under study. Since labeling the described roles of the gene name mentioned in each (gene/protein name, PubMed ID, paragraph) pair is a multi-labeling problem, we computed both the one-label and macro metrics for these (gene/protein name, PubMed ID, paragraph, label) pairs in the performance evaluation. The learning curves of DMLS Step 3 can be found in Figure S1b of the Supplementary File. On the 5-fold validation results, DMLS Step 3 obtains average auROC values of 90.2% and 92.3% in labeling the pairs with ‘target gene’ and ‘TF’ labels, respectively. The corresponding average validation auPRC values are 86.9% and 76.9%, respectively. These one-label performance values lead to the average macro validation auROC/auPRC = 91.3%/81.9% (Figure 3a and b). The macro recall and TNR also reach 77.0% and 88.6%, respectively (Table 2). The macro and one-label auROC, auPRC, recall and TNR values together suggest that DMLS Step 3 can comprehend the described roles of the mentioned gene names in the paragraphs of CRM-related articles. Other performance metrics are provided in Table 2. Since the manually curated ground truths are only available for the summarized target gene and regulatory TF lists of the CRMs under study in each article, the generalization test is deferred to DMLS Step 4.

**Table 2.** The 5-fold validation performance summary of DMLS Step 3 in annotating the (gene/protein name, PubMed ID, paragraph) pairs to have ‘target gene’ and/or ‘TF’ labels on the paragraph 5-fold validation sets

DMLS Step 3	auROC (%)	auPRC (%)	F1 (%)	Precision (%)	Recall (%)	TNR (%)
Target gene labeling validation results	90.2	86.9	78.8	81.6	76.6	87.3
TF labeling validation results	92.3	76.9	70.1	64.7	77.3	90.0
Macro validation results	91.3	81.9	74.5	73.1	77.0	88.6

### DMLS Step 4 precisely summarizes the TF and target gene lists for the CRMs under study in each CRM-related article

**Figure 4.**
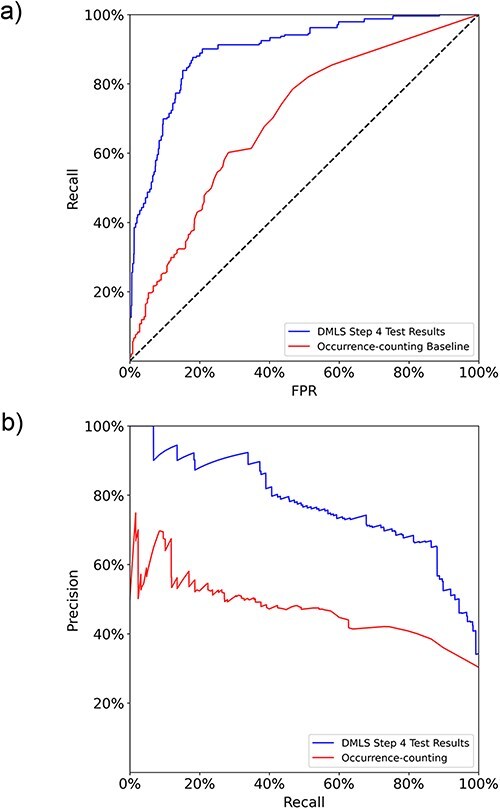
The macro (a) ROC curves and (b) PR curves comparison between DMLS Step 4 and the name-occurrence-counting baseline method on the gene/TF list summary test set.

After the paragraph labeling process, DMLS Step 4 summarizes the labeled paragraphs that contain the same gene name to get the final list of described target genes and regulatory TFs of the CRMs under study in each article. The TFs and genes involved in the CRMs under study are summarized into lists of the form (gene name, PubMed ID, target gene/TF labels). Therefore, the performance evaluation of DMLS Step 4 is carried out on the correctly identified (gene name, PubMed ID, label) tuples. The final aggregation performance of DMLS Step 4 on the prepared gene-summary test set is listed in Table 3. The test macro auROC and auPRC of DMLS in summarizing the described target gene and TF lists are 89.7% and 77.6% (Figure 4), respectively, demonstrating acceptable knowledge extraction performance. The rest of the performance evaluation metrics listed in Table 3 provide a similar conclusion. Notice that the lower precision in summarizing TF lists is partly due to the low prevalence (around only 24.2%) of available regulatory TF lists of CRMs in articles from the test set. One may also observe that the performance of summarizing the target gene lists of CRM-related articles (DMLS Step 4) is better than that of comprehending the gene name-containing paragraphs that describe target genes (DMLS Step 3). One possible reason is that some paragraphs for one gene name may be wrongly comprehended but correctly summarized for the whole article. In summary of the performance evaluation sections on DMLS Step 1, Step 3 and Step 4, each step of DMLS is well-trained and performs well, making DMLS applicable to literature prescreening of newly published biomedical articles.

**Table 3.** The test set performance of DMLS Step 4 in summarizing the target genes and regulatory TFs of the studied CRMs in each article

DMLS Step 4	auROC (%)	auPRC (%)	F1 (%)	Precision (%)	Recall (%)	TNR (%)
Target gene list summary test results	91.8	88.7	83.5	78.7	88.8	83.2
TF list summary test results	87.5	66.5	57.1	42.3	88.1	70.9
Macro test results	89.7	77.6	70.3	60.5	88.5	77.0

### Case study

We provide a real case study in this section to elucidate the usage and results of the DMLS pipeline. Take the article with PubMed ID 30476189 [‘Retrograde BMP signaling activates neuronal gene expression through widespread deployment of a conserved BMP-responsive cis-regulatory activation element’ (20)] as an example. After reading its abstract, DMLS Step 1 identified that this article contains experimental results for modular transcription regulation in *Drosophila*. Since DMLS Step 2 found the full text of this article on the PMC FTP service, the .xml file of its full text was then automatically downloaded. Based on the downloaded .xml file, DMLS Step 3 then parsed the paragraphs of this article and removed paragraphs in the ‘Materials and Methods’, ‘Supplementary Material’, ‘Acknowledgements’, ‘Supplementary Data’ and ‘Reference’ sections. In the parsed paragraphs, the synonyms of different genes/proteins were recognized. Based on the paragraphs that contain at least one gene/protein name, DMLS Step 3 labeled the described gene names as the regulatory TFs or target genes in these paragraphs one by one using the fine-tuned deep paragraph comprehension network. The supporting paragraphs and the identified regulatory TFs or target genes were provided by DMLS Step 3 as a .csv file for user reference. For example, in the second last paragraph of the ‘Result’ section of this example article (Figure 1b), DMLS Step 3 inferred this paragraph, which contains the gene name *sickie*, to describe the experimental results of the gene *sickie*, and labeled this gene as the target gene of the CRMs under study. In a closer manual check, one can see that this paragraph depicts the identification of the regulatory program between *sickie* and the CRM under study based on RNA-seq experiment analysis. The manual inspection confirms the correctness of DMLS Step 3. Finally, DMLS Step 4 summarized the labeling of all gene name-containing paragraphs in this example article and provided the regulatory TF and target gene lists that include (pMad and Medea) and (*cv-2, cg7781, twit* and *sickie*), respectively, for the CRMs under study. As a verification, these DMLS-summarized lists of target genes and TFs were also reported by REDfly curators. Note that DMLS can obtain the target gene and TF results discussed in the full texts in only several minutes, releasing human burdens tremendously.

To better understand the sequence and function annotation of the CRMs investigated in this example article, we utilized regCNN (19), a CRM-identifying tool, to scan the binding site regions of pMad and Medea. When considering the binding sites probed for pMad, regCNN reports that the region located on Chr 2L:19934742…19935553 (genome version: dm6), which contains the pMad ChIP-seq binding peaks (21) and overlaps with the *sickie* gene, is the potential regulatory CRM region. Moreover, this CRM is annotated by CFA (22), a CRM function annotator, to carry out promoter functions. These downstream analyses of the regulatory sequences that recruit pMad and Medea to regulate the target gene *sickie* are similar to the conclusion made by the authors and the REDfly human inference results. From the case study demonstration, DMLS is shown to provide automated transcription literature screening for TFs and target genes of CRMs under study and can identify their relationships when coupled with CRM analysis tools.

### The DMLS tool implementation

We have also implemented an easy-to-use tool to facilitate the usage of the systematic pipeline for extracting the described target genes and regulatory TFs involved in modular transcription regulation from the literature. The data processing and deep learning network prediction in DMLS were based on the programming language Python. DMLS enables batch processing of several selected PubMed abstracts to allow users to construct their own CRM knowledge bases. Furthermore, full-text retrieval from the PMC FTP service was also implemented in the tool to automate the DMLS pipeline. For articles that do not have full texts freely deposited in the PMC database, DMLS can also interpret their abstracts to extract the information in the abstracts. After the execution of the implemented DMLS tool, the information outputs provided in different steps are stored in several .csv files. The DMLS web service can be freely accessed at https://cobis.bme.ncku.edu.tw/DMLS/. In addition, the command-line version of DMLS is also available at https://github.com/cobisLab/DMLS/ for non-commercial usage.

A complete example input file and the corresponding output results can also be found on these websites. In the example, the demo input file consists of several abstracts of *Drosophila* research articles downloaded from the PubMed database. Then, the output result files of this example are also presented on the websites. Users can open the input example to check the required input file format and investigate the output results provided by DMLS through the example output files.

## Discussions

### The selection of the ensemble learning algorithm in DMLS Step 1

In DMLS Step 1, an ensemble model that aggregates the five data-bootstrapping networks was built based on the AdaBoost algorithm. We investigated the robustness of selecting different machine-learning models in this stacking process. In this investigation, we used 3 other well-known machine learning algorithms to obtain the aggregation ensemble model: Random Forest, Support Vector Machine (SVM), and XGBoost. To select the proper hyperparameters, we applied the 5-fold cross-validation and data number learning curve techniques on the article ensemble set for these three learning algorithms. For the SVM algorithm, the final hyperparameters selected were (i) soft margin C = 1 × 10^5^ and (ii) kernel: RBF kernel with $\gamma $ = 0.1. On the other hand, 150 decision trees with max depth = 2 were chosen as the base learners for the Random Forest algorithm. Finally, we set the number of base learners to be 5 (decision stumps with depth = 2), learning rate = 0.3 and regularization $\lambda $ = 0.3 for the XGBoost algorithm. Since the ensemble aggregation algorithm selection is also part of the hyperparameter selection process (11), this investigation was performed based on the average validation results on the ensemble set. The aggregation model using each of the three well-known machine learning methods was ensured to reach convergence between the average training and the average validation learning curves (see Figure S2 in the Supplementary File). We summarized the final average validation results on the ensemble set in Table 4. The performance metrics in identifying articles describing modular transcription regulation using different aggregation algorithms are similar for AdaBoost and Random Forest. Their performance metrics have also been found to be better than those of XGBoost and SVM. Since the boosting methods are generally less prone to overfitting than the Random Forest algorithm, we thus selected the AdaBoost algorithm as the ensemble aggregation method in the final construction of DMLS Step 1.

**Table 4.** The ensemble performance summary of DMLS Step 1 using different ensemble learning algorithms on the article ensemble set

Ensemble algorithm	auROC (%)	auPRC (%)	F1 (%)	Precision (%)	Recall (%)	TNR (%)
AdaBoost	94.4	93.8	86.4	86.8	86.0	87.2
Random Forest	94.6	94.1	87.3	87.6	87.1	87.8
XGBoost	93.7	91.1	87.3	87.6	87.1	87.8
SVM	84.3	92.8	86.6	87.2	86.1	87.4

### Comparison with related works

DMLS is a systematic pipeline developed to help researchers construct their own CRM knowledge bases. It prescreens CRM-related articles and then extracts the described target genes and regulatory TFs of the CRMs investigated in each article. Since DMLS is the first pipeline that can extract the target genes and regulatory TFs involved in modular transcription regulation from the literature, we only compared the corresponding parts of DMLS with well-known simple baseline methods.

Burn *et al*. (23) previously proposed that article classification can be done through sentiment analysis using Convolutional Neural Network (CNN) and Long Short-Term Memory (LSTM) modeling. Therefore, we compared the function of DMLS Step 1 with the CNN and LSTM modeling methods. We re-implemented the CNN and LSTM networks designed by Burn *et al*. and retrained them on the article training-validation set. The detailed network architectures for CNN and LSTM modeling can be found in the work of Burn *et al*. (23) and our previous research (4). In this comparison, we ensured that the hyperparameters were selected to keep the model well-trained and fitted (Figure S3 in the Supplementary File). The final hyperparameters for the CNN and LSTM modeling networks can be found in Section S3 of the Supplementary File. We compared the DMLS Step 1 generalization performance with that of CNN modeling and LSTM modeling (Table 5). On the test set, the auROC and auPRC of DMLS Step 1 (auROC/auPRC = 92.3%/92.2%) outperform the CNN modeling by 0.7% and 0.3%, respectively. DMLS Step 1 achieves much better test performance than the LSTM modeling by at least 6.0% larger in auROC and 7.6% larger in auPRC, indicating the superiority of DMLS Step 1. From these observations, we can conclude that DMLS Step1 can help better leverage the prediction variations for non-CRM-related articles than the CNN and LSTM models.

**Table 5.** The performance comparison among DMLS Step 1, the CNN modeling and the LSTM modeling in identifying articles describing modular transcription regulation experiments on the article test set

Article identification	auROC (%)	auPRC (%)	F1 (%)	Precision (%)	Recall (%)	TNR (%)
DMLS Step 1	92.3	92.2	83.7	84.5	82.8	84.9
CNN modelling	91.6	91.9	83.3	81.6	84.9	80.8
LSTM modeling	86.3	84.6	77.1	74.5	79.8	72.7

Since no other tool can handle the CRM target gene/regulatory TF extraction task, we compared the final summary lists of DMLS Step 4 with the intuitive gene name-occurrence-counting method on the gene-summary test set. The intuitive gene name-occurrence-counting method identifies a (gene/TF name, PubMed ID) item as a summary result if the number of occurrences of the gene/TF name in the article with the specified PubMed ID is larger than a specified threshold. The ROC curve and PR curve evaluation of this baseline method was generated by varying the accepted counting number thresholds. On the other hand, DMLS Step 4 includes only the (gene/TF name, PubMed ID, label) pair as a summary result if at least one of the gene name’s describing paragraphs has its comprehension probability calculated by DMLS Step 3 larger than a specified threshold. As shown in Figure 4, DMLS Step 4 (macro auROC = 89.7%, macro auPRC = 77.6%) outperforms the name-occurrence-counting baseline model (macro auROC = 69.8%, macro auPRC = 47.1%) by 19.9% in macro auROC and 30.5% in macro auPRC on the gene/TF list summary test set. From these results, we verify that DMLS can better summarize the contents of modular transcription regulation than the simple gene name-occurrence-counting method, leading to more confident extracted lists of the described target genes and regulatory TFs of the CRMs under study for a given article.

Previously, we have developed a tool called YTLR (4) that aims to help comprehend yeast transcription-regulation articles. However, genes are regulated in a more switch-like manner in yeast, and YTLR was developed to identify such switch-like transcription regulation-related literature. More specifically, YTLR recognizes yeast TF regulatory articles and TF binding articles and then extracts sentences that describe how a TF regulates/binds to a gene. Meanwhile, transcription regulation is usually carried out in a modular manner for metazoan species. The modular transcription-regulation-related articles mostly only describe how a gene is controlled by CRMs. The TFs that participate in CRM-mediated gene regulation are separately discussed in the articles. What makes the situation difficult is that in the collected 419 CRM-related articles that have free full texts deposited in PMC, only 135 of them directly state the CRM binding TFs in the texts. Therefore, target genes and regulatory TFs of the CRMs under study in the CRM-related articles can only be separately considered, implying that the TF-gene sentence extraction strategy used in YTLR does not apply to metazoan modular transcription-regulation-related articles. Furthermore, due to different literature description styles, YTLR is not readily available for understanding CRM regulation results for metazoan species. For example, using the transcription factor regulation article identification network of YTLR Stage I to extract CRM-related articles on the article test set only resulted in an auROC value of 87.4%. There is around 5% auROC performance degradation in YTLR compared with DMLS Step 1 (auROC = 92.3%, Table 1). For YTLR Stage II, it cannot extract any of the target genes that are involved in the discussed CRMs. To overcome the hindrance, we redesigned the pipeline to fit the need for the comprehension of the CRM-related articles and developed the DMLS tool. The deep learning model in DMLS Step 1 is changed to an ensemble-based architecture, and DMLS Step 3 and Step 4 are dramatically modified from a sentence-based manner (as in YTLR) to a paragraph-based model to enhance the target gene extraction from the full texts. The enhancement of DMLS over YTLR enables DMLS to comprehend CRM-related articles.

Currently, FlyBase (17) bi-monthly updates its manually curated publications that relate to the *Drosophila* genome. Parts of these publications are related to modular transcription *cis*-regulation. Another regularly updated resource of manually curated CRM-related publication summaries is the REDfly (6) database. However, the accumulation of research articles on *Drosophila* outpaces the efforts of human curators. For example, more than 5692 *Drosophila*-related articles were published in 2023. Among them, five articles were curated by REDfly to be CRM-related (REDfly v9.6.4, updated on 21 April 2024). Two additional articles published in 2023 were also deposited in REDfly, but no curated target gene was found in them and thus abandoned in this discussion. These five articles were also curated by the FlyBase team (FB2024_02, released on 23 April 2024). There are far more *Drosophila*-related articles than the curated CRM-related ones. On the other hand, DMLS Step 1 also identifies the five REDfly-curated CRM-related articles. Furthermore, DMLS Step 1 sifts out 638 CRM-related articles from the 5692 articles associated with *Drosophila*. In view of the literature curation speedup, DMLS can broaden the dissemination of modular transcription regulation knowledge from the published papers for the fly community.

### DMLS also shows good performance when trained and tested on randomly partitioned sets

In developing DMLS, the training-validation and test sets were prepared by year-aware partitioning. In the year-aware training-validation and test set partitioning, information on articles before 2014 was put into the training-validation set, while publications later than 2016 were reserved as the test set. The year-aware data partitioning helps evaluate the tool generalization performance across the time course of the writing style shift. As an alternative data preparation step, we re-sampled the ground-truth dataset into the randomly sampled training validation and test sets in each step of DMLS. The performance evaluations on the randomly sampled test sets for DMLS Step 1 and Step 4 are summarized in Tables 6 and 7. As shown in Tables 1 and 6, DMLS Step 1 achieved a slightly better auROC value (auROC = 94.9%) on the randomly sampled datasets than on the year-aware results (auROC = 92.3%). In addition, DMLS Step 4 also obtained a slightly higher macro auROC value (macro auROC = 90.9%, Table 7) on the randomly sampled datasets than on the year-aware datasets (macro auROC = 89.7%, Table 3). Other metrics also reveal similar trends. However, the evaluation of the randomly sampled test sets may have an optimistic estimation of the tool performance on future publications since, in reality, the evolved writing styles of these future publications could not be captured by the training process. The purpose of reserving future writing styles for a fairer evaluation is the main reason for using year-aware partitioning when implementing the DMLS tool. In summary, we demonstrated that DMLS can manifest good performance in CRM-related article identification and target gene/TF extraction on both the randomly partitioned datasets and the year-grouped datasets.

**Table 6.** The DMLS Step 1 (identifying articles describing modular transcription regulation experiments) performance summary on the randomly partitioned test set

DMLS Step 1 (trained on the randomly partitioned dataset)	auROC (%)	auPRC (%)	F1 (%)	Precision (%)	Recall (%)	TNR (%)
Test results	94.9	94.4	87.4	87.0	87.9	86.9

**Table 7.** The performance of DMLS Step 4 (summarizing the target genes and regulatory TFs of the studied CRMs in each article) on the randomly partitioned gene-summary test set

DMLS Step 4 (trained on the randomly partitioned dataset)	auROC (%)	auPRC (%)	F1 (%)	Precision (%)	Recall (%)	TNR (%)
Target gene list summary test results	88.7	83.7	77.5	70.4	86.2	74.7
TF list summary test results	93.1	79.5	67.4	54.6	88.1	80.3
Macro test results	90.9	81.6	72.5	62.5	87.1	77.5

### Limitations of DMLS

The developed DMLS pipeline aims to help users prescreen *Drosophila* articles on a large scale. The final outputs of DMLS include both the described TF/target genes of the CRMs under study and their supporting paragraphs within the scanned articles. Users can check the appended supporting paragraphs in the result files to quickly understand the regulation mechanisms for the described TFs/target genes of the investigated CRMs. However, the detailed regulatory sequence information is usually not directly described in the texts of different research articles. In REDfly, regulatory sequences are additionally inferred from the reported restriction maps by senior researchers or via communication with the original authors. Since DMLS is a natural language processing tool to automate the comprehension of modular transcription-regulation-related articles, the tool itself cannot handle such a type of sequence inference. Nevertheless, the developed DMLS pipeline can be coupled with algorithms [regCNN (19), a CRM-identifying tool, and CFA (22), a CRM function annotator] previously developed by our team to additionally scan the potential sequences and functions of the CRMs under study in the given articles. The sequence extraction and functional annotation of CRMs were demonstrated in the case study. Combining the target gene/TF information, the scanned CRM sequences and annotated CRM functions, the overview of the CRM regulation discussed in the given articles can be briefly and automatically sketched. We plan to integrate all the CRM-related analysis tools developed by our team with DMLS in its future updates.

While REDfly has invested great human resources to try to keep up with the publication pace, the current overarching purpose of DMLS is to lessen the loading of literature curators. In addition, DMLS can also help catch up on articles accidentally missed during the curation process. Hence, DMLS does not intend to replace human curation totally but to accelerate the process with lower costs. The benefits of DMLS are for both database curators and laboratory members who want to build up their own private knowledge repositories. In the current version, DMLS is developed for *Drosophila*. Since articles describing modular transcription regulation can share many similar language patterns, it is expected that the pipeline of deep networks in this research can be transferred to other species. Transfer learning of the deep networks may require a much smaller number of curated publications. To enable the support of DMLS to other species, the species-specific preparation of gene synonyms for gene name recognition and a small number of modular transcription-regulation-related articles for model transfer learning and evaluation are required. Multiple species support will be one of the goals in future updates of DMLS when manual cross-species transcription literature curation is ready. Notice that because our previous work of YTLR encounters the article writing style discrepancy and information incompatibility between switch-liked transcription regulation and metazoan modular *cis*-transcription regulation (see ‘Comparison with related works’ for details), transfer learning does not work for YTLR to fit the task of understanding CRM-related articles. For other metazoan species observed to have modular transcription regulation, DMLS can be fine-tuned using transfer learning to help comprehend CRM-related articles in these species since the article styles are believed to be similar.

Finally, DMLS is a natural language processing tool that helps identify CRM-related articles and comprehend the full texts of the CRM-related articles to extract the involved target genes and TFs of the CRMs under study. In the case study, we showed that DMLS can obtain the results reported by manual curation when considering the full texts of the example article. However, the human experts working for REDfly further carefully investigated the Supplementary Files of the case study article to obtain 13 extra target genes that were not discussed in the full text. The information not discussed in the full texts but only deposited in the Supplementary Files was not interrogated by DMLS. DMLS was designed to help comprehend the full text of CRM-related articles, and the ‘Supplementary’ section and the Supplementary Files are not considered in the process (as stated in the ‘DMLS Step 3: labeling the roles of the described gene names contained in paragraphs of CRM-related articles’ section). Some of the CRM target genes curated by REDfly are discussed in the full texts, while others can only be inferred from the Supplementary Files by domain experts. Although supplementary materials are common sources of additional information, understanding and extracting the data results from the supplementary files still feature a challenging task in the field of artificial intelligence due to the diverse supplementary data formats. Supplementary file formats are diverse, and the data analysis techniques required for understanding the experiment results recorded in the supplementary files are ever-changing. What is worse is that most supplementary files are deposited through different links, and there is no unified way to download these files. Currently, DMLS is designed only for the comprehension of the full texts of CRM-related articles. Automatic data analysis for diverse types of supplementary materials is the future research focus for machine literature understanding, and DMLS will be updated if such artificial intelligence techniques are ready. It is worth noting that because only the target genes and TFs explicitly discussed in the full texts can contribute to actionable data samples for paragraph-based natural language models, the evaluation metrics used for evaluating DMLS Step 3 and Step 4 count only the curated target genes and TFs explicitly discussed in the full texts. These performance evaluations were calculated for the full-text comprehension task.

## Conclusions

In this research, we constructed a systematic pipeline called DMLS to automate the process of literature prescreening for the described target genes and regulatory TFs of CRMs under study in *Drosophila* modular transcription-regulation-related articles. The overall knowledge extraction for the described CRM target gene and regulatory TF lists from CRM-related articles outperforms the simple occurrence-counting baseline method. In order to facilitate the usage of the designed DMLS pipeline, a software tool was implemented for users. Via DMLS, the rampant accumulation of biomedical publications can be prescreened and processed in a batch manner. We believe that the utilization of DMLS coupled with CRM identification and annotation tools can greatly broaden our understanding of metazoan modular transcription regulation.

## Data Availability

The Supplementary File and the source codes for this research can be found at https://github.com/cobisLab/DMLS/ and https://cobis.bme.ncku.edu.tw/DMLS/.

## References

[1] Yang T.-H. (2019) Transcription factor regulatory modules provide the molecular mechanisms for functional redundancy observed among transcription factors in yeast. *BMC Bioinf*., 20, 1–16. 10.1186/s12859-019-3212-8 PMC6933673 31881824

[2] Hardison R.C. and Taylor J. (2012) Genomic approaches towards finding cis-regulatory modules in animals. *Nat. Rev. Genet*., 13, 469–483. 22705667 10.1038/nrg3242 PMC3541939

[3] Hua J.T. , Ahmed M., Guo H. et al. (2018) Risk SNP-mediated promoter-enhancer switching drives prostate cancer through lncRNA PCAT19. *Cell*, 174, 564–575. 30033362 10.1016/j.cell.2018.06.014

[4] Yang T.-H. , Wang C.-Y., Tsai H.-C. et al. (2022) YTLR: extracting yeast transcription factor-gene associations from the literature using automated literature readers. *Comput. Struct. Biotechnol. J*., 20, 4636–4644. 36090812 10.1016/j.csbj.2022.08.041 PMC9449546

[5] Björne J. , Ginter F., Pyysalo S. et al. (2010) Complex event extraction at PubMed scale. *Bioinformatics*, 26, i382–i390. 20529932 10.1093/bioinformatics/btq180 PMC2881365

[6] Rivera J. , Keränen S.V.E., Gallo S.M. et al. (2019) REDfly: the transcriptional regulatory element database for Drosophila. *Nucleic Acids Res*., 47, D828–D834. 30329093 10.1093/nar/gky957 PMC6323911

[7] Li G. , Ross K.E., Arighi C.N. et al. (2015) miRTex: a text mining system for miRNA-gene relation extraction. *PLoS Comput. Biol*., 11, e1004391. 10.1371/journal.pcbi.1004391 PMC4583433 26407127

[8] Vlachos I.S. , Paraskevopoulou M.D., Karagkouni D. et al. (2015) DIANA-TarBase v7.0: indexing more than half a million experimentally supported miRNA:mRNA interactions. *Nucleic Acids Res*., 43, D153–D159. 25416803 10.1093/nar/gku1215 PMC4383989

[9] Arighi C.N. , Lu Z., Krallinger M. et al. (2011) Overview of the BioCreative III workshop. *BMC Bioinf*., 12, 1–9. 10.1186/1471-2105-12-S8-S1 PMC3269932 22151647

[10] Yang T.-H. , Wang C.-Y., Tsai H.-C. et al. (2021) Human IRES Atlas: an integrative platform for studying IRES-driven translational regulation in humans. *Database*, 2021, baab025. 10.1093/database/baab025 PMC8094437 33942874

[11] Abu-Mostafa Y.S. , Magdon-Ismail M. and Lin H.-T. (2012) *Learning from Data*. Vol. 4. AMLBook, New York, NY.

[12] Laza R. , Pavón R., Reboiro-Jato M. et al. (2011) Evaluating the effect of unbalanced data in biomedical document classification. *J. Integr. Bioinform*., 8, 105–117. 10.2390/biecoll-jib-2011-177 21926440

[13] Yang T.-H. , Liao Z.-Y., Yu Y.-H. et al. (2023) RDDL: a systematic ensemble pipeline tool that streamlines balancing training schemes to reduce the effects of data imbalance in rare-disease-related deep-learning applications. *Comput. Biol. Chem*., 106, 107929. 10.1016/j.compbiolchem.2023.107929 37517206

[14] James G. , Witten D., Hastie T. et al. (2013) *An Introduction to Statistical Learning*. Vol. 112. Springer, New York City, NY.

[15] Yang T.-H. (2021) An aggregation method to identify the RNA meta-stable secondary structure and its functionally interpretable structure ensemble. *IEEE/ACM Trans. Comput. Biol. Bioinform*., 19, 75–86. 10.1109/TCBB.2021.3082396 34014829

[16] Lee J. , Yoon W., Kim S. et al. (2020) BioBERT: a pre-trained biomedical language representation model for biomedical text mining. *Bioinformatics*, 36, 1234–1240. 31501885 10.1093/bioinformatics/btz682 PMC7703786

[17] Larkin A. , Marygold S.J., Antonazzo G. et al. (2021) FlyBase: updates to the Drosophila melanogaster knowledge base. *Nucleic Acids Res*., 49, D899–D907. 33219682 10.1093/nar/gkaa1026 PMC7779046

[18] Pan S.J. and Yang Q. (2009) A survey on transfer learning. *IEEE Trans. Knowl. Data Eng*., 22, 1345–1359.

[19] Yang T.-H. , Yang Y.-C. and Tu K.-C. (2022) regCNN: identifying Drosophila genome-wide cis-regulatory modules via integrating the local patterns in epigenetic marks and transcription factor binding motifs. *Comput. Struct. Biotechnol. J*., 20, 296–308. 35035784 10.1016/j.csbj.2021.12.015 PMC8724954

[20] Vuilleumier R. , Lian T., Flibotte S. et al. (2019) Retrograde BMP signaling activates neuronal gene expression through widespread deployment of a conserved BMP-responsive cis-regulatory activation element. *Nucleic Acids Res*., 47, 679–699. 30476189 10.1093/nar/gky1135 PMC6344883

[21] Hammal F. , de Langen P., Bergon A. et al. (2022) ReMap 2022: a database of Human, Mouse, Drosophila and Arabidopsis regulatory regions from an integrative analysis of DNA-binding sequencing experiments. *Nucleic Acids Res*., 50, D316–D325. 34751401 10.1093/nar/gkab996 PMC8728178

[22] Yang T.-H. , Yu Y.-H., Wu S.-H. et al. (2023) CFA: an explainable deep learning model for annotating the transcriptional roles of cis-regulatory modules based on epigenetic codes. *Comput. Biol. Med*., 152, 106375. 10.1016/j.compbiomed.2022.106375 36502693

[23] Burns G.A. , Li X. and Peng N. (2019) Building deep learning models for evidence classification from the open access biomedical literature. *Database*, 2019, baz034. 10.1093/database/baz034 PMC6449534 30938776

